# Characterisation of Early-Life Fecal Microbiota in Susceptible and Healthy Pigs to Post-Weaning Diarrhoea

**DOI:** 10.1371/journal.pone.0169851

**Published:** 2017-01-10

**Authors:** Samir Dou, Pascale Gadonna-Widehem, Véronique Rome, Dounia Hamoudi, Larbi Rhazi, Lyes Lakhal, Thibaut Larcher, Narges Bahi-Jaber, Arturo Pinon-Quintana, Alain Guyonvarch, Isabelle L. E. Huërou-Luron, Latifa Abdennebi-Najar

**Affiliations:** 1 UP 2012.10.101.EGEAL, Institut Polytechnique LaSalle Beauvais, rue Pierre Waguet Beauvais, France; 2 INRA, UR1341 ADNC, Domaine de la Prise, Saint-Gilles, France; 3 Institut polytechnique LaSalle Beauvais, Direction de la recherche, rue Pierre Waguet Beauvais, France; 4 INRA, UMR 703 APEX, Ecole Nationale Vétérinaire Agroalimentaire et de l’Alimentation Nantes-Atlantique (Oniris), Nantes, France; 5 INVIVO-NSA, Direction Scientifique Technique et Innovation, Département Innovation et Prospectives, Talhouët Saint Nolff, France; Max Rubner-Institut, GERMANY

## Abstract

Early-life microbial exposure is of particular importance to growth, immune system development and long-lasting health. Hence, early microbiota composition is a promising predictive biomarker for health and disease but still remains poorly characterized in regards to susceptibility to diarrhoea. In the present study, we aimed to assess if gut bacterial community diversity and composition during the suckling period were associated with differences in susceptibility of pigs to post-weaning diarrhoea. Twenty piglets from 5 sows (4 piglets / litter) were weaned in poor housing conditions to challenge their susceptibility to post-weaning diarrhoea. Two weeks after weaning, 13 pigs exhibited liquid faeces during 2 or 3 days and were defined as diarrhoeic (D) pigs. The other 7 pigs did not have diarrhea during the whole post-weaning experimental periodand were defined as healthy (H) pigs. Using a molecular characterisation of fecal microbiota with CE-SSCP fingerprint, Next Generation Sequencing and qPCR, we show that D and H pigs were mainly discriminated as early as postnatal day (PND) 7, i.e. 4 weeks before post-weaning diarrhoea occurence. At PND 7 H pigs displayed a lower evenness and a higher abundance of *Prevotellaceae*, *Lachnospiraceae*, *Ruminocacaceae* and *Lactobacillaceae* compared to D pigs. The sPLS regression method indicates that these bacterial families were strongly correlated to a higher Bacteroidetes abundance observed in PND 30 H pigs one week before diarrhoea. These results emphasize the potential of early microbiota diversity and composition as being an indicator of susceptibility to post-weaning diarrhoea. Furthermore, they support the health promoting strategies of pig herds through gut microbiota engineering.

## Introduction

Environmental and maternal bacteria quickly colonize offspring gut after birth and shape the onset of a healthy intestinal immune system and its future development [1]. Impaired gut microbiota composition during the neonatal period may lead to chaotic growth of species after weaning, suggesting a greater permissiveness to pathogen colonisation and induction of pro-inflammatory status [2,3]. This is especially relevant in swine production with each farm microbial environment being different and possibly impacting animal health status and the productive outcome. It has been shown that maternal diet or antibiotic treatment may induce a long lasting impact on the establishment of gut microbiota, gut biology and growth performances of offspring pigs [4–6]. However, none of these studies have associated the early-life fecal microbiota diversity and composition to the further suceptibility of post-weaning diarrhea in pigs.

Alpha diversity and evenness of the bacterial community structure have been associated with the stability over time and the invasibility of the ecosystem.[7,8]. Zhang *et al*. [9] showed that genetically immunodeficient mice displayed a higher bacterial evenness, probably due to lack of selective pressures. A restricted microbial exposure during the first days of life reduced the abundance of beneficial bacteria such as *Lactobacillus* and increased expression of pro-inflammatory cytokines in pigs [2]. More recently, Mach *et al*. [10] showed that the *Prevotella* enterotype of adut pigs which was correlated to higher production of secretory immunoglobulin A than the *Ruminococcaceae* enterotype, can be predicted from the abundance of *Clostridium* cluster XIVa and *Lactobacillus* in 14 days-old pigs. All together, these data make the early gut microbiota of great importance for the future pig health.

In the present study, we aimed to assess if gut bacterial community diversity and composition during the suckling period were associated to differences in susceptibility of pigs to post-weaning diarrhoea. By using 16S rRNA gene approaches coupled with capillary electrophoresis single-stranded conformation polymorphism (CE-SSCP), quantitative polymerase chain reaction (qPCR) and complementary cultivation-based analysis on fecal microbiota of pigs we measured bacterial ecological parameters (alpha diversity, beta diversity, and evenness indices) of the fecal bacterial community across ages upon the onset diarrhoea in a challenging post-weaning environment. As diversity and evenness parameters discriminated piglets at postnatal day (PND) 7 according to the further occurrence of post-weaning diarrhoea, the sequence analysis of 16S rRNA was performed at this early stage to determine Operational Taxonomic Units (OTUs) that discriminated pigs according to their susceptibility or resistance to post-weaning diarrhoea. Moreover, PICRUSt pipeline was applied on the 16S rRNA sequencing data and the imputed relative abundances of KEGG pathways in each respective sample were used to predict alterations in pig fecal microbiome functions.

## Material and Methods

### Ethical statement

The experimental protocol was designed in compliance with the guidelines for animal research of the French Ministry of Agriculture and all other applicable national and European guidelines and regulations for experimentation with animals (http://www2.vet-lyon.fr/ens/expa/acc_regl.html). The current experiment was conducted in preclinical facilities of LaSalle Institute that received the agreement from the French veterinary services to conduct animal Research (LaSalle facilities agreement number C60-200-001, delivered in 2013). Due to the fact that the experimental study was conducted before the official national requirements of ethical evaluation established on 1 February 2013 (Article R.214-118 the Rural Code and Maritime Fishing and the Decree of 1 February 2013 on the ethical evaluation), it was not evaluated by an official ethical committee. To note Ethical committee of LaSalle Beauvais and many French Institutes were created after 2013 (May 2013 for LaSalle Institute). The protocol was conducted on supervision of Dr Thibaut Larcher from the French veterinary school of Nantes (UMR703 PAnTher) who assists the scientific and technical staff of LaSalle Beauvais during animal euthanasia and tissue collection in compliance with the relevant laws and institutional guidelines.

### Animals and experimental design

A total of 5 multiparous sows (Large-white x Landrace) and their litters (Large-white x Landrace x Pietrain) from the experimental research center of INRA (Saint-Gilles, France) were used. The experiment was conducted in one batch. The litters were selected regarding the rank parity of sows (from 3 to 5) and the litter size. Sows received the same standard diet for gestating and lactating periods. Interlitter adoption was not allowed. Initially, all newborn piglets of the 5 litters were weighed. At PND 1, 4 pigs (2 males, 2 females) per litter (n = 20 pigs) were selected based on their liveweight at birth and their growth during the first 24 h of life as an indicator of colostrum intake.

Before weaning, sow-reared piglets had no access to creep feed. Althought introduction of creep feed during suckling period is a common practice to improve growth performances, access to solid feed before weaning was avoided in order to strengthen the challenging effect of food transition on gut health. Antibiotic treatments on the selected sows and offspring were not allowed along the experiment. At weaning (PND 21), selected piglets were together transported to another experimental facility (LaSalle Institute, France) and raised in poor housing conditions to challenge their susceptibility to digestive disorders. Poor housing conditions were achieved by leaving the pens voluntarily dirty and room temperature was 20°C instead of the recommended 25°C [11–13]. The 4 pigs from each litter were allocated in the same 3 m^2^ pen. Pigs were fed a starter diet (Zenith Flore made by *Evialis* company) free of any growth-promoting additive.

### Colostrum and feces sampling

Two milliliters of colostrum were collected during the first hour after the first piglet birth from each sow. A pool of colostrum from the 4 median teats of each sow was sampled. Half of collected colostrum was immediately frozen at -20°C until immunoglobulin (Ig) analysis. The remaining colostrum was centrifuged at 400 g for 12 min at 4°C to collect the aqueous phase which was stored at -20°C until TGFβ1 assay. Colostrum intake was estimated according to the weight gain of newborn pigs over the first 24 hours of live [14].

Body weight of selected pigs was measured at birth, PND 1, 7, 14 and 21 and weekly after weaning **(S1 Fig)**. Post-weaning daily weight gain was calculated by dividing the weight gain over around one week by the number of days that separate two consecutive weight measurements (around 7 days). Faeces were collected at PND 7, 14, 21, 30, 38 and 47 on an individual basis and the dried matter (DM) content was assessed at PND 30, 38 and 47. During the lactation period, no diarrhoea was observed on the selected pigs. After weaning, clinical signs of diarrhoea were individually monitored on a daily basis. PND 38 was the first day when diarrhea occurred. Pigs were defined as diarrhoeic (D) pigs according to the occurrence of liquid faeces during 2 to 3 consecutive days. Pigs exhibiting solid faeces during the whole post-weaning experimental periodwere denoted as healthy H) pigs. Liquid and solid faeces were *a posteriori* defined according to the dried matter (DM) content, less than 20% of DM for liquid faces and more than 20% of DM for solid faeces. The distribution of D and H pigs within each litter is depicted in **S1 Table.** At PND 50, pigs were slaughtered by exsanguination after deep anesthesia with Pentobarbital treatment (30 mg/Kg). Immediately, after opening the intestinal cavity, a 5 cm segment of proximal colon was collected, rinsed with cold phosphate buffered saline and immediately frozen in dry ice for later genomic DNA analysis.

### Colostral immunoglobulin G and A and TGF-B1 assays

Concentration of Ig was quantified using swine IgG or IgA ELISA Quantitation Kit (Bethyl Laboratories, Montgomery, Texas, USA). Samples were diluted in Tris buffered saline containing 1% BSA 0.05% Tween-20 as described [15]. The bioactive form of Transforming Growth Factor Beta 1 (TGFβ1) was quantified using the TGFβ1 Emax ImmunoAssay System (Promega Corporation, Madison, USA) according to manufacturer’s instructions. The concentration of IgA, IgG and TGFβ1 was multiplied by the estimated amount of ingested colostrum to calculate the quantity of ingested IgG, IgA and TGFβ1 during the first 24 h of life.

### Microbiological analyses

#### DNA extraction

After the mechanical lysing of bacteria using the Lysing Matrix E kit (MP Biomedical), from 150 mg of faeces, DNA extraction was performed using the Stool Mini Kit (Qiagen). The yield and purity of DNA extracts were quantified using a Nanodrop 2000 (Thermo Fisher Scientific) and DNA quality was tested on 1.5% agarose gel electrophoresis.

#### qPCR assay on 16S rRNA genes

16S rDNA copy numbers for total bacteria, *Firmicutes*, *Bacteroidetes*, *Lactobacillus* and *Enterobacteriaceae* were estimated by quantitative PCR (qPCR) with the RotorGene6000. Briefly, each reaction tube contained 3 μL DNA, 4 μL Evagreen Super Mix (SolyBiodyn) and 250 nM primers (S2 Table), and reaction mix was brought up to 20 μL with high grade molecular biology water. The reaction tubes were placed in a thermocycler and the polymerase was activated for 12 min at 95°C. The PCR cycles were: 30 s for denaturation at 95°C, 30 s for annealing at 60°C and 30 s for elongation at 72°C. To ensure that cycle of quantification (Cq) values were in the linear range of the standard curve, qPCR was performed on 10-fold serial dilutions of the DNA extracts. Standard quantitation curves of primers for total bacteria and *Enterobacteriaceae* were obtained from pure culture of *Escherichia coli*. Standard quantitation curve of primers for *Lactobacillus*, Firmicutes and Bacteroidetes were assessed with *Lactobacillus sp*., *Fecalibacterium prausnitzii* and *Bacteroides fragilis*, respectively. The Cq value of each sample was compared to the linear standard curve and plotted as Cq vs. gene log copies number [16]. Results are expressed as the number of targeted copies per 100 μg of DNA [17,18].

#### Culture-dependent analysis of *Enterobateriaceae*

Six pigs were ramdomly selected before diarrhoea at PND 30, PND 38 and PND 47 for culture-dependent quantitation of *Enterobactericeae* in feces. Immediately after sampling, 5 g of feces were diluted 10% (w/v) in Trypton salt (1 g / L Trypton, 8.5 g / L NaCl, 0.5 g / L chlorhydrate cystein) under anaerobic conditions in an anaerobic chamber and homogenized with a stomacher. Ten-fold serial dilutions were made anaerobically and each serial dilution was plated in duplicate on different culture media. *Enterobacteriaceae* were counted by plating 1 mL into Plat Count Violet Red Bile Glucose medium, following manufacturer instructions (Biokar diagnostic), respectively. Petri dishes were incubated at 37°C. *Enterobacteriaceae* abundance are CFU per gram of fecal dried matter.

#### CE-SSCP profile processing

Age-related evolution of the microbial diversity was evaluated from PND 7 to PND 38 in D and H pigs. Twenty five μL volume PCR reactions were performed with high fidelity AmpliTaq Gold 360 (Life technologies), 60 ng DNA and 300 nM universal primers (S2 Table) for the target region V3 of 16S rDNA of bacteria [19]. The PCR products were diluted 10X in high grade formamide (Life Technologies) and heated for 10 min at 95°C. The denatured amplicons were immediately placed on ice for 10 min [20]. Then, 1 μL was mixed to 0.15 μL internal lane size standard (Life Technologies) and 10 μL formamide. Capillary electrophoresis was performed on an ABI prism sequencer 3130 with 6% CAP polymer (Life Technologies), 10% glycerol (Sigma Aldrich), 10% Tris-Borate-EDTA (Sigma Aldrich) and high grade molecular biology water. The running voltage was 15 KV at 25°C. The electrophoresis provided a fingerprint pattern per animal that represents a picture of the fecal bacterial ecosystem. Within the fingerprint pattern, dominant, subdominant and very small peaks were associated with the numerical dominant, subdominant and rare phylotypes (Sequences or Operational Taxonomic Units, OTUs), respectively [21] (S2 Fig). The structure of fecal bacterial community fingerprints provided by Capillary Electrophoresis-Single Strand Conformational Polymorphism (CE-SSCP) were analysed with Stat fingerprints [22], a CRAN (Comprehensive R Archive Network) package on R (Version.2.14.1). Firstly, the molecular fingerprints were aligned by the internal standards Genscan Lyse (500, Life Technologies). Secondly, the common baseline between the CE-SSCP profiles was defined. Finally, the fingerprints were normalised to a defined baseline. The fingerprint is composed by discrete peaks that represent at least one 16S rRNA sequence (so-called phylotype or ribotype) [21].

Statfingerprint package enables the calculation of two indices of alpha diversity (Simpson and Shannon indices). The alpha diversity is an index that summarises the fecal bacterial community assemblage in terms of number of phylotypes (richness) and their relative abundances (evenness) [23]. To compare the dissimilarity of bacterial community structure between pigs in terms of shared phylotypes (beta diversity), CE-SSCP fingerprints were transformed into binary profiles (presence/absence of phylotypes) and the distance of dissimilarity was computed with Jaccard index [24,25].

#### Sequencing analysis and microbial bioinformatics analysis

Sequencing analysis was performed on fecal DNA extracted from PND 7 piglets. The V3-V4 region of the 16S rRNA coding gene was amplified with primers in S2 Table [26]. Amplicons were purified with magnet beads (AMPure XP Beckman Coulter Genomics) and the concentrations of samples were normalized to 4 nM using a fluorometer Qbit 3.0 (Life Technologies). Amplicon were tagged, pooled and sequenced with the MiSeq instrument (Illumina) that produced two demultiplexed reads of the amplicons from 5’ and 3’ ends.

Both generated reads were merged with the paired-end assembler PandaSeq, providing maximum 500 bp length sequences [27]. The pool of sequences was firstly cleaned with Mothur package by keeping only sequences with less than 8 homolymers, no ambiguous base, and of 300 to 500 bp length. Chimeric sequences were further deleted using Usearch algorithm with Quantitative Insights Into Microbial Ecology (Qiime) software [28–30]. *De novo* OTU clustering and taxonomic assignement were performed on Green Gene databases (Version 05/2013). Only OTUs with more than 3 sequences were considered as valid. Finally, the OTU data were submitted to PICRUSt analysis to predict the metagenome of fecal bacterial communities from KEGG (Kyoto Encylopedia of Genes and Genomes) data bases [31].

### Genetic susceptibility to Enterobacteria-associated diarrhoea, expression of MUC13 A and MUC13 B alleles

Mucin 13 genotype was shown to be a strong genetic marker of *Enterobacteriaceae*-associated diarrhoea [32]. The pig genotype of the mucin 13 (MUC13) gene that governs the susceptibility to *Enterobacteriaceae* adhesion and Enterobacteria-induced diarrhoea, was characterised. Homozygous pigs for MUC13 A are healthy to Enterobacteria-associated diarrhoea while susceptible animals carry at least one MUC13 B allele [33]. Using the Trizol chloroform procedure, genomic DNA was extracted from colon tissues sampled at slaughter (PND 50). Briefly, after bead beating and Trizol chloroform treatment, DNA was precipitated with 100% ethanol and washed twice with 0.1 mM sodium citrate and 10% ethanol washing solution. Finally DNA was dissolved in the NaOH solution buffered with HEPES to pH 7–8 until performing PCR amplification of the MUC13 gene. The region of intron 2 of MUC13 gene was amplified with following primers F7/R7 (MUC13-F: 59-TTC TAC TCT GAT TCC ACA TCA CG-39; MUC13-R: 59-TGG TCA TGT CTA GGA CTC TTT GAG-39). PCR were made with the FIREPOL Master Mix (7.5 mM MgCl2), 200 nM of MUC13-F and MUC13-R and 1**μ**l of pig genomic DNA. MUC13 amplification was processed in the thermal cycler PTC-100 (MJ Research) after enzyme activation for 5 min à 95°C. Thirty cycles of amplification were performed (denaturation at 95°C for 40 s, annealing step at 63°C for 60 s and elongation step at 72°C for 40 s) and a final elongation step at 72°C for 5 min. The MUC13 A allele that is associated to resistance to diarrhoea is sized 151 pb. The MUC13 B allele that is associated to susceptibility to diarrhoea is sized 83 pb [33].

### Statistical analysis

Statistics were carried out with R Core Team (Version.2.14.1; R: A language and environment for statistical computing; R Foundation for Statistical Computing, Vienna, Austria. URL http://www.R-project.org/). The effect of age on microbial diversity and evenness within each group was analysed using repeated measures ANOVA with a paired Student t-test. At each age group comparisons were done with student t-test or Mann-Whitney test when distributions of data within each group were normal or non-normal, respectively. The normality was tested with Shapiro and Wilk test [34]. Correlation between *Enterobacteriaceae* abundance and fecal dry matter (DM) was evaluated using Pearson’s test. The prevalence of MUC13A and MUC13B alleles in D and H groups was evaluated using Pearson’s Chi-squared test.

Significance of Jaccard distance between D and H was assessed with ANOSIM statistical test to evaluate the distances at 999 permutations between pig groups [35]. ANOSIM R-values range between -1 and 1, with the value 0 indicating completely random grouping. R-values ranged between 0 and 0.25 indicate a barely separation, between 0.25 and 0.5 a moderate separation, and between 0.5 and 0.75 a good separation; significance occurred at P < 0.05.

For microbiota characterisation by sequencing method, only OTUs with a variance > 10^−8^ were taken into account (the table of raw OTUs contained 5589 OTUs and were reduced to 608 OTUs). OTU count were normalized with a scaling factor method [36]. The differential abundance of OTUs count was assessed with the exact test assuming negative binomial distribution [37]. False Discory Rate (FDR) adjustement was applied on raw p-values [38].

A supervised classification method, sparse Partial Least Square Discriminant Analysis (sPLDA), was used to estimate the contributive OTUs in H vs. D classification [39]. This method performs a variable (OTUs) selection with Lasso penalization method [39]. Iterative cross validation method allowed selecting the most stable OTUs in the model of classification. An initial tuning of sPLS-DA parameters was performed to determine the main OTUs and number of components that enable discrimination of H and D pigs as early as PND 7 with the lower possible error rate. It selected 99, 43 and 43 OTUs for components 1, 2 and 3, respectively **(S3.1, S3.2 and S3.3 Table)**. However, the assessment of the model performance by cross validation revealed an average error rate of 50% **(S3.4 Table)**. A second sPLS-DA with the initial sPLS-DA OTUs that were systematicaly included across all folds (64 discriminant OTUs, i.e. 63 OTUs for component 1 and 1 OTU for component 2, with stability of feature over all crossvalidations = 1) indicated that combining these stable features led to the lowest mean error rate (in average 11%; **S3.5 Table**). These discriminant OTUs clustered within the second sPLS-DA plotted dependently on post-weaning susceptibility to diarrhoea.

Contributor OTUs to H and D groups were correlated to predicted KEGG pathways provided by PICRUSt analysis [31]. Finally, univariate analysis (exact test) between H and D groups, was also performed on the predicted metagenomic data provided by PICRUSt.

## Results

### Characterization of post-weaning diarrhoea

During the period between between PND 38 and 42, 13 pigs had diarrhoea and were considered as diarrhoeic (D) while the 7 other ones had no digestive disorders and were considered as healthy (H). Among litters (n = 5), 3 were strongly affected by diarrhoea with 3 D pigs out of 4, while in the 2 remaining litters only 1 pig out of 4 presented diarrhoea. Differences in intake of colostral immune factors and in genetic MUC 13 allele frequencewere evaluated in order to rule out the hypothesis that these factors were involved in the prevalence of post-weaning diarrhea. Colostrum intake and that of immune colostral components were not different between D and H **(S4 Table)**. Pearson’s Chi-squared test did not show any significant correlation between MUC13 genotype and the susceptibility to post-weaning diarrhoea (P > 0.05; **S3 Fig**).

**Fig 1:**
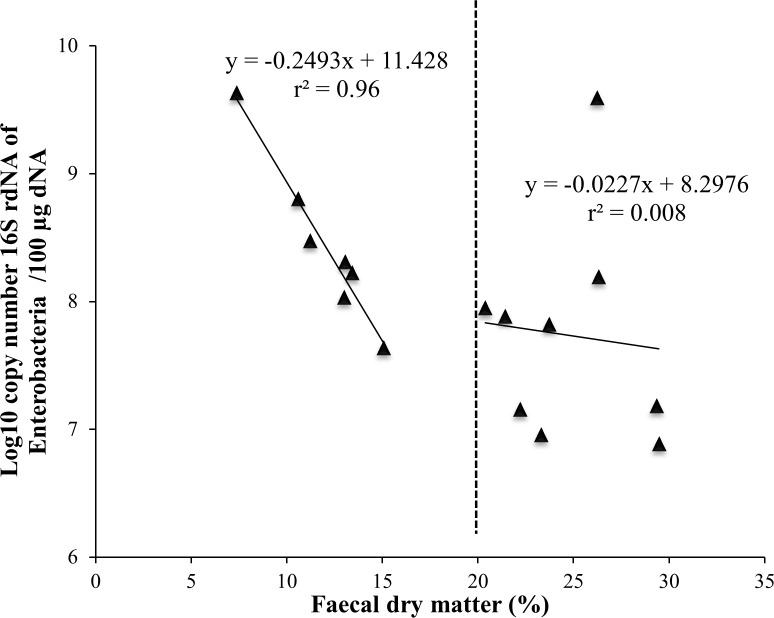
*Enterobacteriaceae* copy number was highly correlated to fecal DM in diarrhoeic (D) pigs (fecal DM < 20%) but not in non-diarrhoeic (H) pigs (fecal DM > 20%) at PND 38.

At PND 38, when diarrhoea occurred, a negative correlation between the fecal DM and the abundance of *Enterobacteriaceae* determined by qPCR was observed within D pigs (when < 20% DM, r^2^ = 0.96, P < 0.001), but not in non-diarrhoeic) H pigs (when > 20% DM, r^2^ = 0.008, P > 0.05) **(Fig 1)**. The culture–dependent quantitation of fecal *Enterobacteriaceae* showed the same negative correlation with fecal DM at PND 38. Significant correlations were not observed before (PND 30) and after (PND 47) diarrhoeic episodes (**S4 Fig).** Quantitative PCR analysis corroborated the higher abundance (P < 0.05) of *Enterobacteriaceae* in D pigs compared to H ones at PND 38 **(S4 Table)**. The increased in *Enterobacteriaceae* was associated with a lower abundance of Bacteroidetes in D pigs at PND 38 (P <0.05) **(S4 Table)**. Differences in *Enterobacteriaceae* were also observed at PND 30. Finally, no significant differences in Firmicutes, Bacteroidetes, Lactobacillus and *Enterobacteriaceae* abundances were observed at PND 7, 14 and 21 **(S4 Table)**.

### Bacterial molecular fingerprint and diversity across ages in H and D pigs

**Fig 2:**
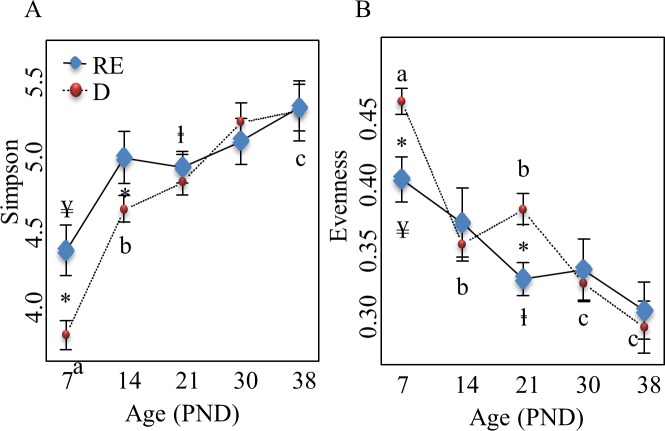
Dynamics of bacterial diversity (A) and evenness (B) in D and H pigs. (A) * indicates significant differences between D and H pigs at each age, P < 0.05; a, b, c indicate significant differences between ages in D pigs, P < 0.05; ¥,ⱡ indicate significant differences between ages in H pigs, P < 0.05. (B) * indicates significant differences between D and H pigs at each age, P < 0.05; a, b, c indicate significant differences between ages in D pigs, P < 0.05; ¥, ⱡ indicate significant differences between ages in H pigs, P < 0.05. H, healthy; D, diarrhoeic.

Simpson diversity index determined with the CE-SSCP method, increased from PND 7 up to PND 21 in H pigs, when it increased from PND 7 up to a maximal value at PND 38 in D pigs (**Fig 2A**). Lower values of Simpson index were observed at PND 7 and PND 14 in D compared to H pigs (**Fig 2A**). Time course analysis of the evenness index revealed its decrease between PND 7 and PND 21 in both groups and till PND 38 in D pigs (P < 0.0001) (**Fig 2B**). Higher values of evenness index were observed at PND 7 and PND 21 in D pigs compared to H pigs (P < 0.01) **(Fig 2B).** No variation through ages was observed for the richness index (data not shown). In addition, the Shannon index was higher in D than in H pigs at PND 21 (data not shown).

**Fig 3:**
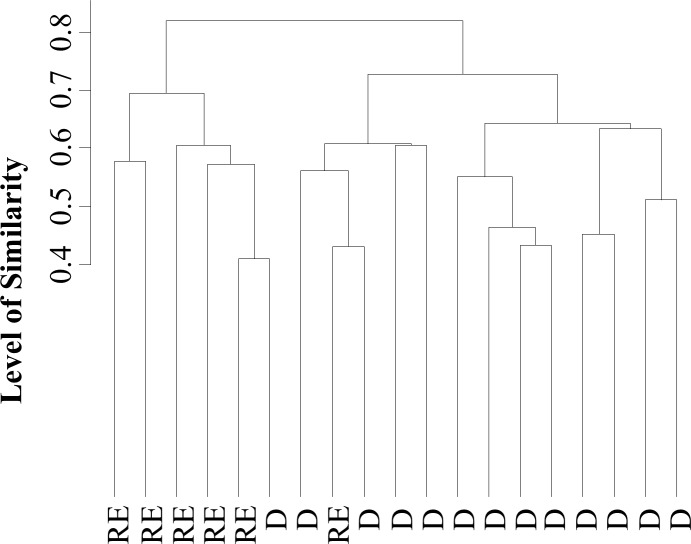
Ascendant hierarchical clustering based on differences of bacterial community structure between D and H pigs at PND 7. Binarized CE-SSCP profiles of animals were compared using Jaccard similarity index and ANOSIM statistical test was applied to assess the significance of the clustering. H, healthy; D, diarrhoeic.

The ascendant hierarchical clustering based on the presence and absence of phylotypes in the CE-SSCP fingerprints moderately discriminated D from H pigs at PND 7 (r = 0.37, P < 0.0001; **Fig 3**) while no more differences were noticed afterwards (data not shown).

### Discriminant bacterial populations at PND 7 between H and D pigs

**Fig 4:**
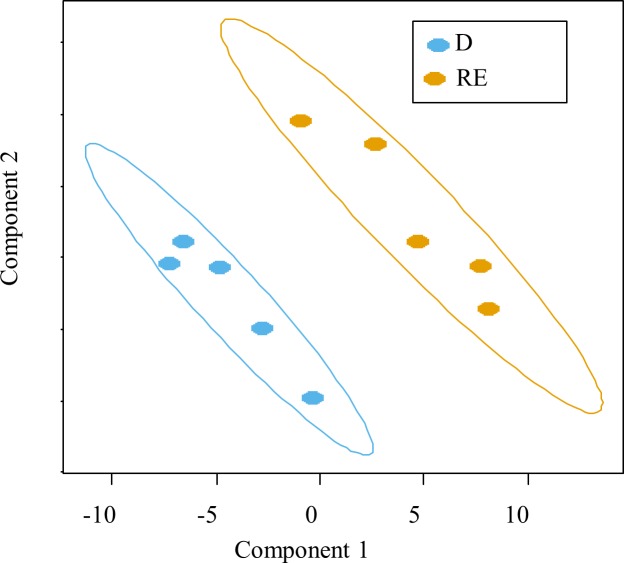
Individual plot of sPLS-DA classification model. Diarrhoeic, D; H, healthy.

**Fig 5:**
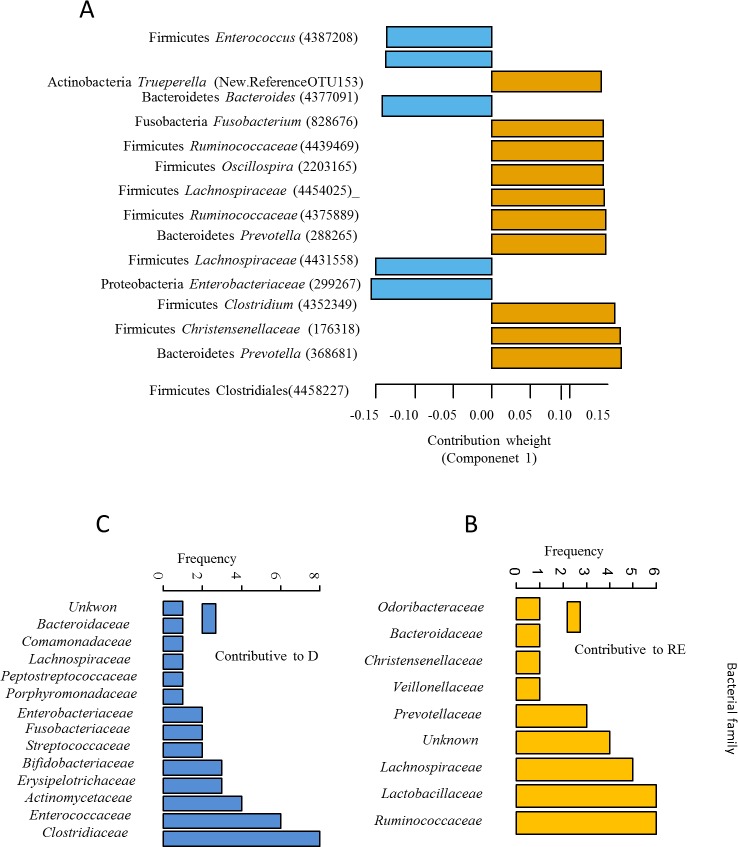
Contribution plots. (A) OTUs in PND 7 pig feces that mostly contributed to group discrimination and their frequency. OTUs are ordered (top 15 OTUs) according to their contribution weight to the component 1 of the second sPLS-DA. H group in Orange bars and D group in blue bars. (B) The frequency of bacterial families among the contributor OTUs in H group (Orange bar). (C) The frequency of bacterial families among the contributor OTUs in D group (Blue bar). Diarrhoeic, D; H, healthy.

sPLS-DA, a supervised classification method, was applied to decipher the most accurate OTUs that contributed to H vs D group discrimination at PND 7 **(Fig 4; S3.6 Table)**. At the phylum level, the discriminating OTUs that contributed the most to differences between PND 7 pig groups belonged to Firmicutes and Bacteriodetes in both H and D groups, but also in Actinobacteria, Proteobacteria and Fusobacteria in D pigs (**Fig 5A** and **S3.6 Table)**. The most frequent discriminant bacterial families were *Ruminococcaceae*, *Lactobacillaceae* and *Lachnospiraceae* in H group (**Fig 5B**) and *Clostridiaceae*, *Enterococcaceae* and *Actinomycetaceae* in D group (**Fig 5C**).

An univariate analysis was performed to determine the differiential abundance of discriminant OTUs between H and D pigs at PND 7. At the family level the differences in bacterial abundance between H and D groups were within *Prevotellaceae* (19% of all discriminant OTUs), *Lachnospiraceae* (12.6%) and *Ruminococcaceae* (11.1%) (FDR-P-Value < 0.05; **S5 Table**). There was also an increased abundance of *Prevotella* (23%) and *Lactobacillus* (6%) genera in H group (**S5 Table**) Among the identified genera with the highest range of abundance changes (>|6.3| log FC), *Prevotella* (5.6 to 7.7 log FC corresponding to 8 OTUs) and *Succiniclasticum* (6.3 log FC corresponding to 1 OTU) were significantly greater and *Fusobacterium* (-8.0 log FC corresponding to 1 OTU) and *Corynebacterium* (-6.4 log FC corresponding to 1 OTU) were significantly lower in H vs. D groups (**S5 Table)**.

### Imputed bacterial functional activity of H and D pigs at PND 7 (PICRUSt analysis)

**Fig 6:**
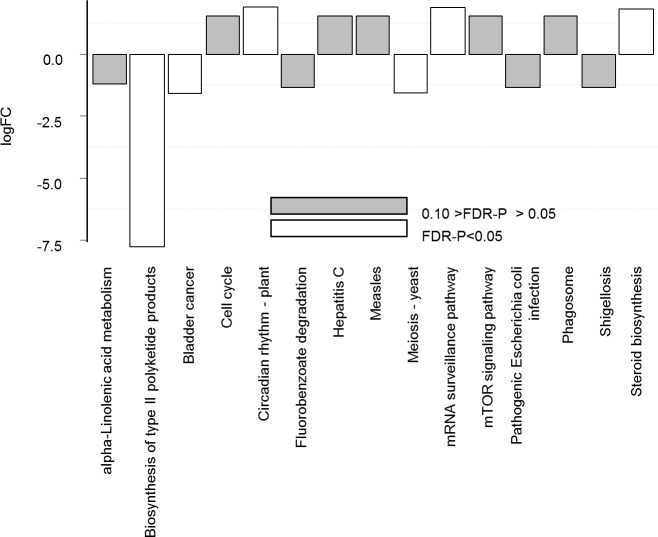
Diffential abundances of imputed bacterial functional activity (KEGG pathways) in H pigs compared to D pigs at PND 7, using PICRUSt analysis. Differential abundance is expressed as log (FC) comparing H to D groups. P-values were adjusted Benjamini-Hochberg P value (white bar, significant at FDR-P < 0.05; grey bar, tendancy at P < 0.10).

PICRUSt was applied on the 16S rRNA sequencing data and the imputed relative abundances of KEGG pathways in each respective sample were used to predict alterations in fecal microbiota functions between PND 7 D and H pigs **(S6 Table)**. At PND 7, H group showed in particular, a significant lower expression of pathways related to host biosynthesis of type II polyketide products, but a higher expression of mRNA surveillance pathway and steroid biosynthesis than in D groups (FDR-p-value <0.05; **Fig 6** and **S6 Table**). Interestingly functions related to pathogenic *Escherichia coli* infection and Shigellosis was less abundant in H compared to D fecal microbiota (FDR-P-Value = 0.05; **Fig 6** and **S6 Table**).

**Fig 7:**
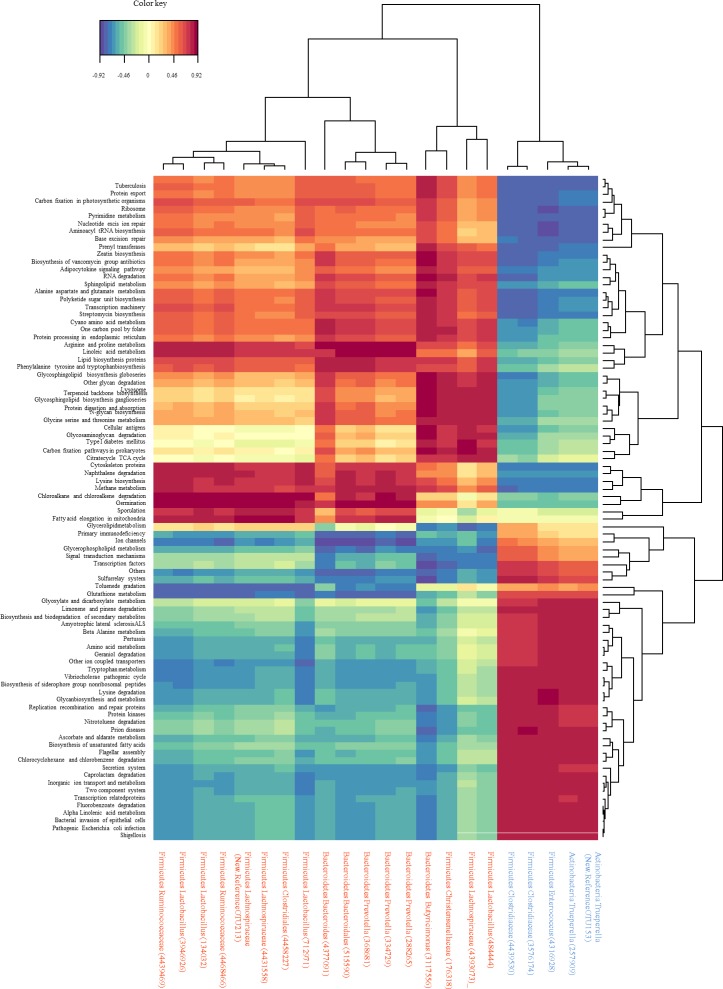
Heatmap of canonical correlations between discriminant OTUs in H (Orange) or D (Blue) groups and predicted KEGG pathways (Level 3) at PND 7. All detailed correlation values are given in S7 Table.

Finally we calculated correlation values between all the discriminant OTUs deciphered by sPLS-DA (listed in **S3.6 Table**) and the expression of functional pathways (**Fig 7** and **S7 Table**). In D microbiota discriminant OTUs were posively correlated to KEGG pathways related to infectious diseases (pathogenic *E*.*coli* and Shigellosis), genetic inflammation processing, cell motility, membrane transport, lysine degradation and beta-alanine metabolism (**Fig 7** and **S7 Table**). In H microbiota discriminant OTUs were positively correlated to antibiotic, lysine biosynthesis, amino acid (arginine, proline, serine, glycin, threonine, alanine, aspartate, and glutamate) metabolism, and methane metabolism (**Fig 7** and **S7 Table**).

### Association between PND 7 discriminant OTUs and post-weaning parameters

**Fig 8:**
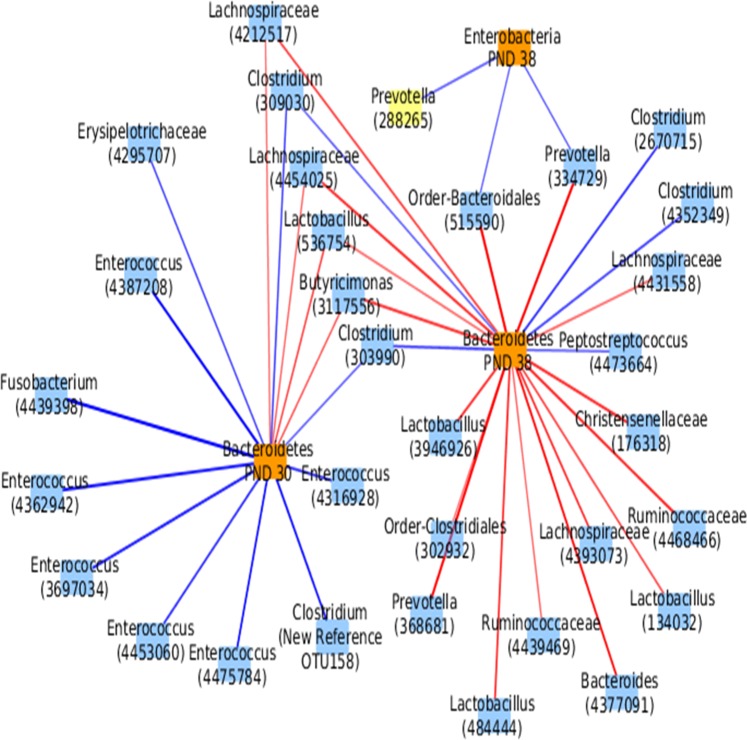
Relevant network of sPLS regression between abundances of Bacteroidetes at PND 30 and PND 38 and *Enterobacteriaceae* at PND30, and discriminant OTUs at PND 7. The threshold of coefficient of correlation used was > |0.5|. Green and red edges indicate negative and positive correlations, respectively. All detailed correlation values are given in S8 Table.

A sPLS regression analysis was used to provide a linear relationship between PND 7 discriminant OTUs and the abundance of total microbiota, Firmicutes, Bacteroidetes, *Enterobacteriaceae* and *Lactobacillus* (evaluated by qPCR) at different time periods (PND 14, 21, 30, 38 and 47). The network of sPLS regression showed that the abundance of Bacteroidetes at both PND 30 and PND 38 were positively associated to several OTUs contributor to H group which belonged to Prevotella, *Lachnospiraceae*, *Ruminococcaceae* and *Lactobacillus* (**Fig 8, S8 Table**). The abundance of *Enterobacteriaceae* at PND 30 was negatively associated to 3 OTUs from the Bacteroidetes phyla. No correlation was highlighed with *Enterobacteriaceae* at PND 38 above the chosen threshold (|Coefficient of correlation| > 0.5).

## Discussion

There is clear evidence that gut microbiota play an important role in driving host metabolism and health [40,41]. However, how early changes in gut microbial colonization contribute to the susceptibility to post-weaning diarrhea is still an unsolved issue. The aim of the current study was to bring new insights in the potential contribution of early gut microbiota in the modulation of health and diseases in later life. More specifically we aimed to draw inferences about relationships between early dynamics of fecal microbiota and occurrence of post-weaning diarrhoea in pig husbandry. The data show that 1- the diversity and evenness indices of fecal bacterial community at PND 7 and their changes over time were different in pigs according to their subsequent susceptibility to post-weaning diarrhoea; 2- the discriminant OTUs at PND 7 that the most contributed to differences in the fecal microbiota taxonomic composition between H and D groups mainly belonged to Firmicutes and Bacteriodetes phyla. Taken together, our data show that specific changes in microbial ecology and presumably in its functional activity occur early in life and that these changes are associated with subsequent susceptibility of pigs to post-weaning diarrhea.

### The early discriminant bacterial population between susceptible and healthy pigs to post-weaning diarrhoea

The structure of the fecal bacterial community of D and H pigs differed as early as PND 7 with a decrease in the relative proportion of dominant bacterial species in D pigs as indicated by the lower diversity and the higher evenness indices. Concern has been raised in ecological theory about the possible link between diversity and evenness of bacterial ecosystems and their ability to respond to gastrointestinal perturbations [42]. Diversity and microbial composition may change permissiveness of the gut ecosystem to pathogen colonization [43]. Stecher *et al*. [44] showed that both the ecosystem with a low complexity and the abundance of commensal bacteria closely related to pathogenic species, could reduce colonisation resistance against pathogens. A higher evenness index has been reported in immunodeficient mice lacking of mature lymphocytes compared to wild-type mice, probably due to the lack of selective pressure on bacterial community in immunodeficient mice [9]. Using in vitro ecosystem analysis Werner *et al*. [45] clearly demonstrated that the evenness index is a good indicator of microbial stability and lowering it down was associated with ecosystem instability in case of abiotic aggression. Therefore in our study a higher evenness in early life associated with its decrease immediately after weaning in D pigs may reflect a lower robustness ecosystem towards secondary bacterial colonisers and a disturbance of the bacterial community complexity at PND 7.

Sequencing analysis of the fecal microbiota at PND 7 revealed that OTUs that mainly discriminated H and D pigs were within the two most abundant phyla of suckling pigs, Firmicutes and Bacteroidetes [10]. The abundance of *Lachnospiraceae*, *Ruminococcaceae* and *Prevotellaceae* families was increased whereas that of *Fusobacteriaceae* and *Corynebacteriaceae* families was deeply decreased in H pigs compared to D pigs. At the genus level the higher abundance of *Roseburia*, *Prevotella* and genera belonging to *Ruminococcaceae* may provide benefits to the host because species from these genera are adapted to metabolize a wide range of complex oligosaccharides and polysaccharides. Indeed *Roseburia* and *Prevotella* are major contributors in the metabolic network of carbohydrate utilization and production of short chain fatty acids [46]. Moreover, *Blautia* and *Prevotella* genera and Bacteroides order were negatively associated with *Escherichia coli*-induced enteric infection in the human colon [47], probably in relation with the susceptibility of the pathogen to short chain fatty acids [48]. Interestingly, the predicted metagenomic KEGG pathways related to pathogenic *Escherichia coli* infection and Shigellosis are enriched in D groups. The abundance of *Enterobacteriaceae* and *Fusobacterium* was positively correlated to intestinal chronic inflammatory diseases [49]. Moreover the predictive pathway involved in the biosynthesis of type II polyketide products was sharply lowered in H group. These organic compounds have a strong antimicrobial activity and in addition, their coding genes are mainly hosted by Actinobacteria phylum and *Escherichia coli* species [50,51]. The relative abundance of polyketide synthases is strongly associated to *Escherichia coli* overgrowth, initiation of inflammation, and intestinal inflammatory bowel diseases [52]. Altogether, the higher abundance of *Lachnospiraceae*, *Ruminococcaceae* and *Prevotellaceae* family in healthy pigs to later post-weaning diarrhea may provide a higher energy harvesting in suckling H piglets and an adequate prevention strategy for pathogen infection. In a more speculative manner, our results may suggest an earlier colonization of mucosal-associated bacterial families that have more efficient abilities for glycan digestion (as in H group) would prepare the intestine and its microbiota to cope with a weaning diet rich in complex carbohydrates as suggested by studies of Koenig *et al*. [53] and Vaishampayan *et al*. [54].

### Bacteroidetes and *Enterobacteriaceae* abundances after weaning may prevent diarrhoea

Major microbiota communities, the dominant phyla, Firmicutes and Bacteroidetes, and two genera, *Enterobacteriaceae* and *Lactobacillus*, were assessed by qPCR in pig feces after weaning to achieve a broader view of the bacterial abundance that may potentially be affected differently in H and D groups [55,56]. Post-weaning Bacteroidetes and *Enterobacteriaceae* abundances were higher in H and D pigs, respectively. An early low bacterial diversity associated with a delayed Bacteroidetes colonization was shown to correlate to a reduced Th1 response in infants born by caesarean section and members of Bacteroidetes phylum are known to interact with the gut immune system, suggesting a link between early microbiota colonization and gut immune maturation after weaning [57]. Additionally *Prevotella* and, in a less extent, *Bacteroides* represent the major genera in Bacteoroidetes phylum in weaned pigs [10]. Their great relative abundance observed after weaning has been related to their capacity to produce enzymes that degrade plant polysaccharides [58,59]. Therefore the higher abundance of Bacteroidetes may allow H pigs to better adapt to post-weaning dietary conditions. The greater *Enterobacteriaceae* abundance in D pigs at PND 30, one week prior to diarrhea, may prefigure the occurrence of diarrhoea a few days later. Further investigations are however needed to establish a causal link between the abundance of Bacteroidetes and *Enterobacteriaceae* during an eubiosis state and the further diarrhoea state.

### Suggested links between early-life bacterial community composition and late Bacteroidetes abundance

The sPLS regression analysis revealed that the abundance of genera from *Prevotellaceae*, *Lachnospiraceae* and *Ruminococcaceae* families at PND 7 were positively correlated to the higher level of Bacteroidetes and were negatively correlated to the lower level of *Enterobacteriaceae* observed in H pigs post-weaning. Some studies suggested a long-lasting conditioning of gut microbiota by the early-life microbiota diversity and composition. Hence, Mach *et al*. [10] have suggested a deterministic evolution of gut microbiota towards *Ruminococcaceae* or *Prevotella* enterotypes after weaning depending on the abundance of *Lactobacillu*s and *Clostridium* cluster XIVa during the suckling period. Jakobsson *et al*. [60] showed that the reduced microbial diversity in neonates born by caesarian section can alter the colonization of Bacteroidetes. Although the long-lasting ecosystemic impact on microbiota by early-life bacterial community remains to be clarified, our study sustains the association between early *Ruminococcaceae*, *Prevotellaceae* and *Lachnospiraceae* abundances and the late Bacteroidetes growth.

## Conclusion

This study is the first that has addressed the relationship between the early fecal microbiota and the further susceptibility to post-weaning diarrhoea in pigs. Our results show that it possible to discriminate differentially susceptible pigs to post-weaning diarrhoea through their early-life bacterial diversity and evenness indices and microbiota composition. Differences in predictive functions associated with changes in bacterial composition may suggest a greater permissiveness to *Enterobacteriaceae* in pigs susceptible to post-weaning diarrhoea. Large scale experiments including several environmental factors would be interesting to improve knowledge about early host-microbiota interactions and the occurence of digestive troubles after weaning. That would open new avenues to the development of novel strategies that could be applied early after birth to prevent further post-weaning digestive disorders.

## Supporting Information

S1 Fig Experimental design. Four piglets/litter and five litters were selected. At postnatal day (PND) 21, piglets were weaned and transferred to another experimental unit and raised in poor housing conditions to challenge their susceptibility to digestive disorders. Pigs were *a posteriori* divided into 2 groups, diarrhoeic (D) and healthy (H) pigs, according to their susceptibility to post-weaning digestive disorders.
(TIF)

S2 Fig Example of a CE-SSCP profile. The area of discrete peaks (in red) represents the relative abundance of different phylotypes. Their number assesses the richness. The relative abundance of peaks and the richness are used to assess the diversity and evenness of the fecal bacterial community.
(TIF)

S3 Fig Expression of MUC13 genes in colon of D and H pigs. The PCR amplification of the Indel region in intron 2 of MUC13 resulted in an amplicon size of 151 or 83 bp for MUC13 A and MUC13 B, respectively. Diarrhoeic, D; H, healthy.
(TIF)

S4 Fig Culture-dependent quantitation of *Enterobacteriaceae*. Correlation between fecal *Enterobacteriaceae* abundance (CFU/g of fecal dried matter) and the percentage of fecal dried matter at PND 30 (a), PND 38 (b) and PND 47 (c).
(TIF)

S1 Table Occurence of H and D pigs per litter. (XLSX)

S2 Table Sequences of primer sets. (PDF)

S3 Table sPLS-DA classification models of H and D pigs. Assessment of performances of the first sPLS–DA classification model (S3.1, S3.2, S3.3 and S3.4 Table) and the second sPLS–DA classification model with the most stable OTUs from the first sPLS-DA (S3.5 and S3.6 Table). Diarrhoeic, D; H, healthy.
(XLSX)

S4 Table Mean (parametric t test) and median (non-parametric Mann-Whitney test) differences between H and D pigs. Tested variables were body weight, daily weight gain, colostrum intake, food efficiency, bacterial diversity (Simpson index) and evenness (Equitability index), and abundance of Lactobacillus, Enterobacteria, Firmicutes and Bacteroidetes at different ages (PND, 14, 21, 30, 38). Diarrhoeic, D; H, healthy.
(XLSX)

S5 Table Differential OTU abundances between H vs D groups using the Univariate Exact-test (R package edgeR) analysis. Diarrhoeic, D; H, healthy.
(XLSX)

S6 Table Differential abundance of predicted KEGG pathways. Imputed bacterial functional activity in H pigs compared to D pigs at PND 7 (PICRUSt analysis).
(XLSX)

S7 Table Canonical correlations between discriminant OTUs in H or D groups and predicted KEGG pathways at PND 7. Positive and negative correlations with |Coefficient of correlation| > 0.5 were highlighted in red or green, respectively.
(XLSX)

S8 Table Correlation matrix of sPLS regression between abundances of Bacteroidetes at PND 30 and PND 38 and *Enterobacteriaceae* at PND30, and discriminant OTUs at PND 7. The corresponding heatmap is presented in Fig 8.
(XLSX)

## Abbreviations

CE-SSCP: Capillary Electrophoresis-Single Strand Conformational Polymorphism
Cq: Cycle of quantification
DM: Dry Matter
KEGG: Kyoto Encyclopedia of Genes and Genomes
NGS: Next Generation Sequencing
qPCR: quantitative polymerase chain reaction
sPLS-DA: sparse Partial Least Square Discriminanat Analysis
OTU: Operational Taxonomic Unit
