# Supplementary figures and images for: Characterisation of Early-Life Fecal Microbiota in Susceptible and Healthy Pigs to Post-Weaning Diarrhoea
Source: PLoS One. 2017 Jan 10;12(1):e0169851. doi: 10.1371/journal.pone.0169851 (PMC5225014; doi:10.1371/journal.pone.0169851)

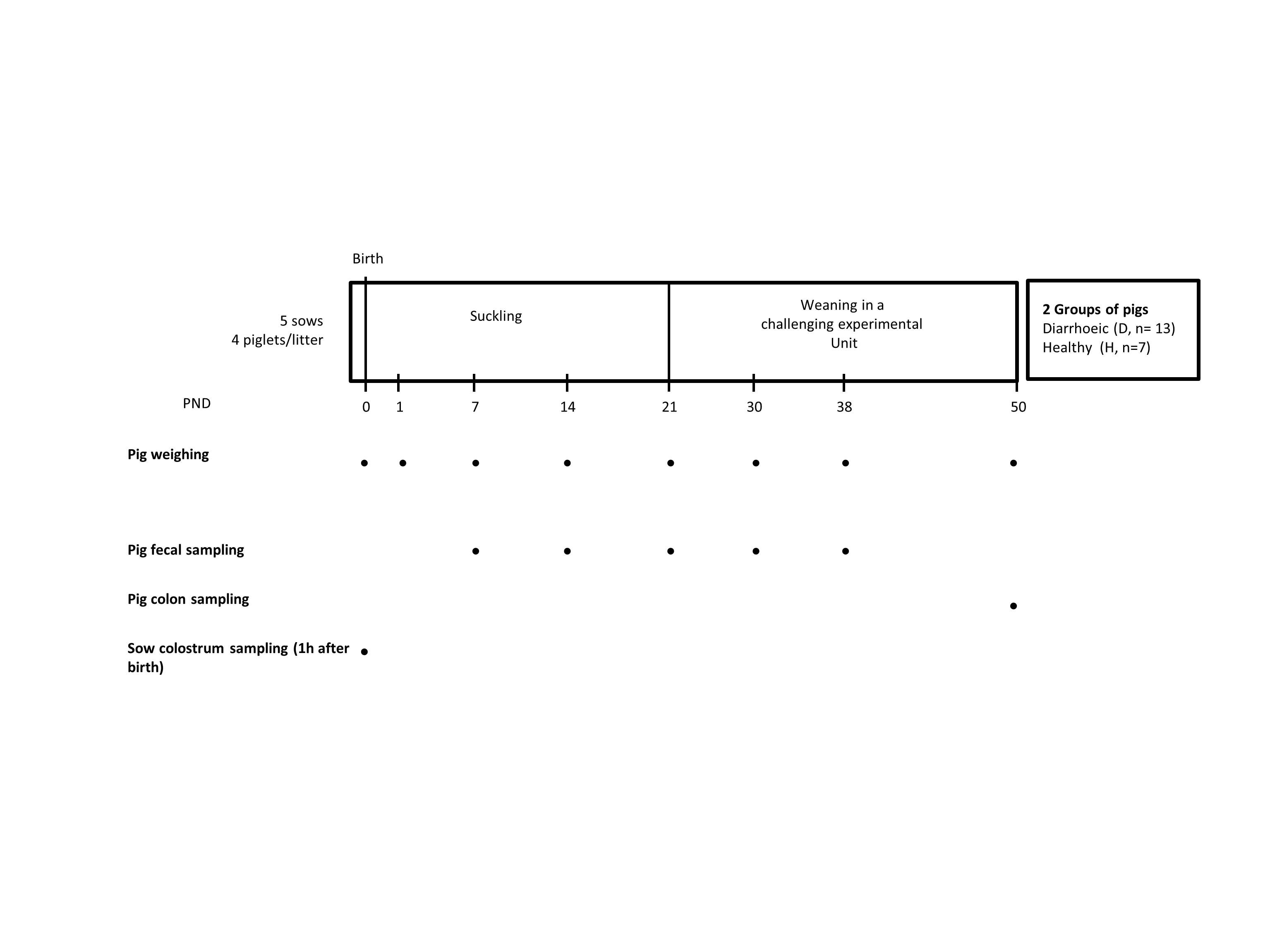

Supplement: S1 Fig — Four piglets/litter and five litters were selected. At postnatal day (PND) 21, piglets were weaned and transferred to another experimental unit and raised in poor housing conditions to challenge their susceptibility to digestive disorders. Pigs were a posteriori divided into 2 groups, diarrhoeic (D) and healthy (H) pigs, according to their susceptibility to post-weaning digestive disorders. (TIF) [file pone.0169851.s001.TIF]

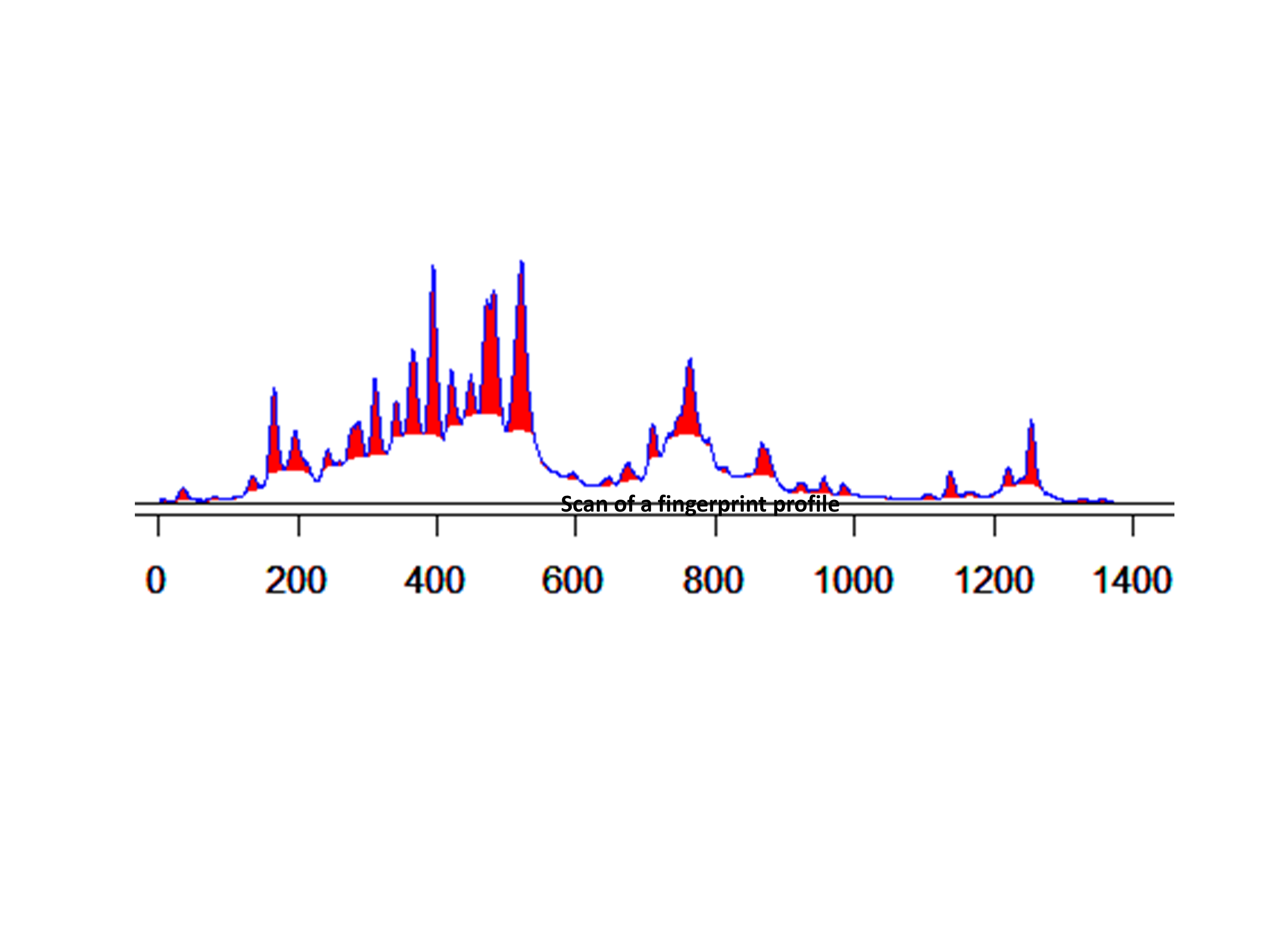

Supplement: S2 Fig — The area of discrete peaks (in red) represents the relative abundance of different phylotypes. Their number assesses the richness. The relative abundance of peaks and the richness are used to assess the diversity and evenness of the fecal bacterial community. (TIF) [file pone.0169851.s002.TIF]

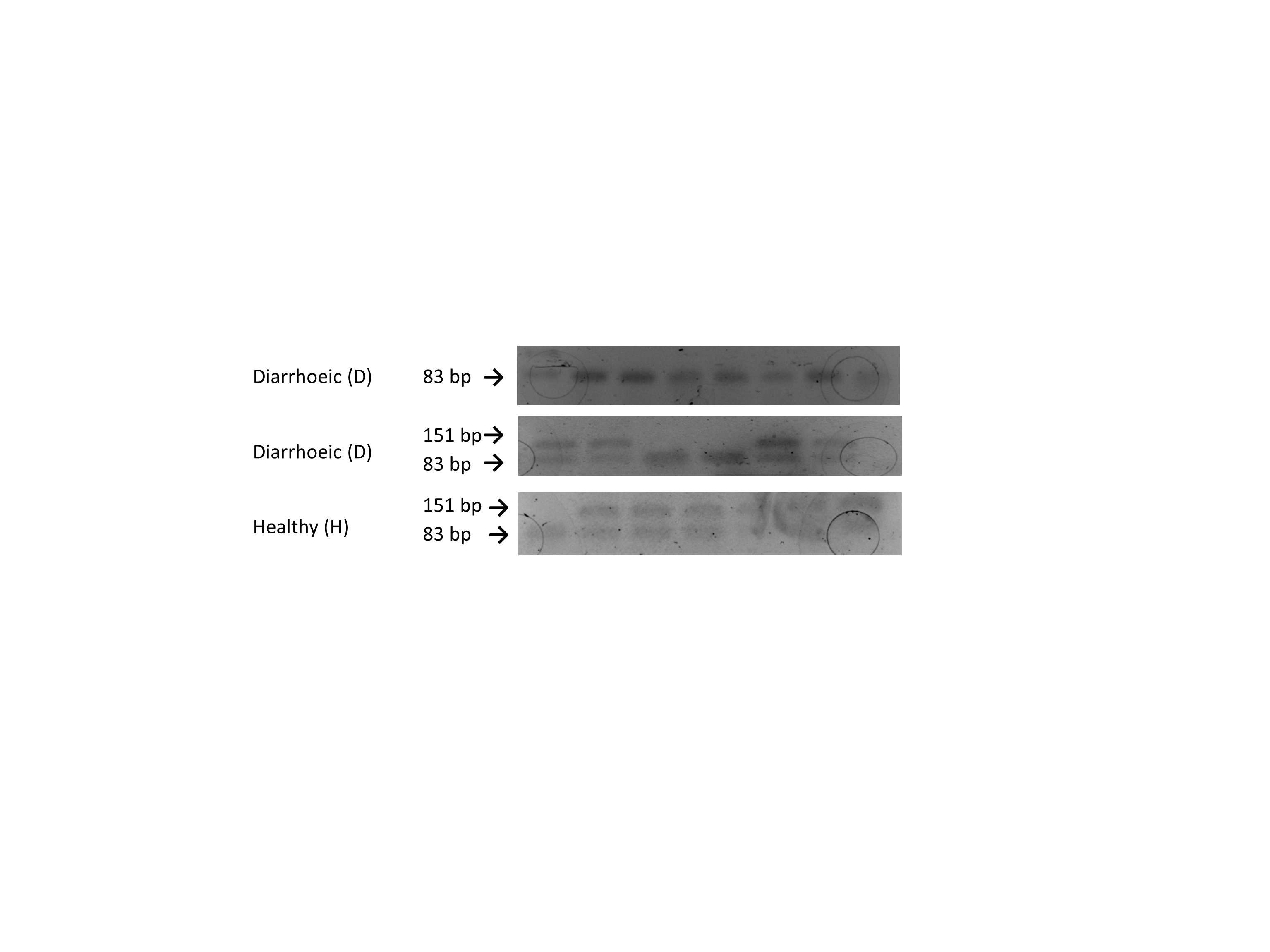

Supplement: S3 Fig — The PCR amplification of the Indel region in intron 2 of MUC13 resulted in an amplicon size of 151 or 83 bp for MUC13 A and MUC13 B, respectively. Diarrhoeic, D; H, healthy. (TIF) [file pone.0169851.s003.TIF]

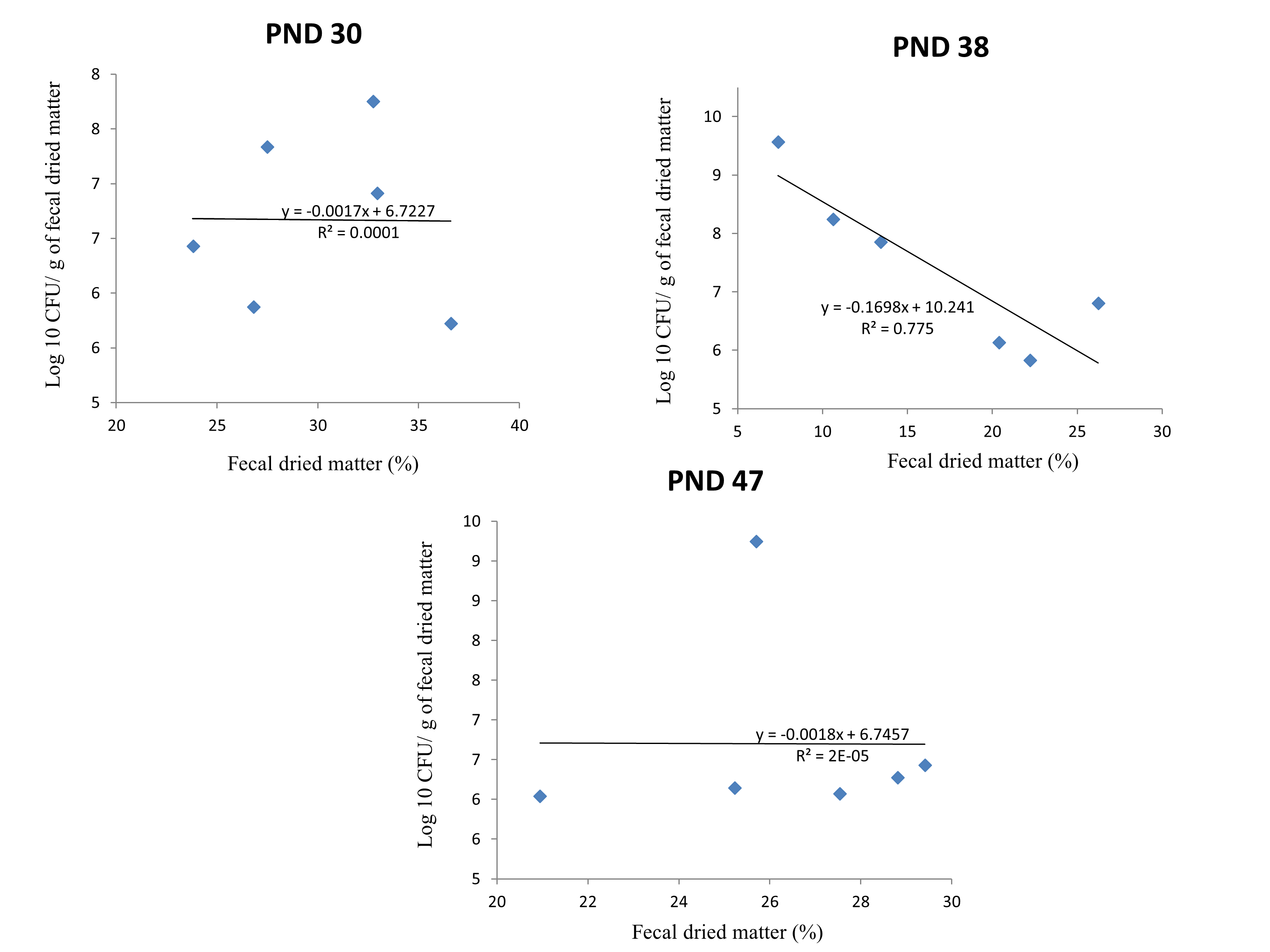

Supplement: S4 Fig — Correlation between fecal Enterobacteriaceae abundance (CFU/g of fecal dried matter) and the percentage of fecal dried matter at PND 30 (a), PND 38 (b) and PND 47 (c). (TIF) [file pone.0169851.s004.TIF]
