# Supplementary material for: Characterisation of Early-Life Fecal Microbiota in Susceptible and Healthy Pigs to Post-Weaning Diarrhoea
Source: PLoS One. 2017 Jan 10;12(1):e0169851. doi: 10.1371/journal.pone.0169851 (PMC5225014; doi:10.1371/journal.pone.0169851)
Supplement: S2 Table — (PDF) [file pone.0169851.s006.pdf]

Table S1: Sequences of primer sets

Targets	Sequences	Sources
CE-SSCP		
Eubacteria	AGGTCCAGACTCCTACGGG<br>6FAM-TACCGCGGCTGCTGGCAC	Pissavin et al. (2012) [19]
Sequencing (MiSeq©)		
Eubacteria	TCGTCGGCAGCGTCAGATGTGTATAAGAGACAGCCTACGGGNGGCWGCAG<br>GTCTCGTGGGCTCGGAGATGTGTATAAGAGACAGGACTACHVGGGTATCTAATCC	Klindworth et al. (2013) [26]
qPCR		
Eubacteria	TCCTACGGGAGGCAGCAGTG<br>TTACCGCGGCTGCTGGCACG	Ettreiki et al. (2012) [17]
Enterobacteria	CATTGACGTTACCCGCAGAAGAA<br>CGCTTGCACCCTCCGTATTA	
Lactobacillus	AGCTTCGAATTAAACCACATGCT<br>TGCCGGAGCTAACGCATT	Pouillart et al. (2010) [18]
